# Teachers’ monitoring of students’ text comprehension: can students’ keywords and summaries improve teachers’ judgment accuracy?

**DOI:** 10.1007/s11409-018-9187-4

**Published:** 2018-12-10

**Authors:** Jan A. A. Engelen, Gino Camp, Janneke van de Pol, Anique B. H. de Bruin

**Affiliations:** 1 Welten Institute, Open University of The Netherlands, Heerlen, Netherlands; 2 Present Address: Department of Communication and Cognition, Tilburg University, Tilburg, Netherlands; 3 Department of Education, Utrecht University, Utrecht, Netherlands; 4 Department of Educational Development and Research, Maastricht University, Maastricht, Netherlands

**Keywords:** Teacher judgments, Metacognitive monitoring, Reading comprehension, Self-regulated learning, Keyword generation, Cue diagnosticity

## Abstract

We investigated intra-individual monitoring and regulation in learning from text in sixth-grade students and their teachers. In Experiment 1, students provided judgments of learning (JOLs) for six texts in one of three cue-prompt conditions (after writing delayed keywords or summaries or without a cue prompt) and then selected texts for restudy. Teachers also judged their students’ learning for each text, while seeing - if present - the keywords or summaries each student had written for each text, and also selected texts for restudy. Overall, monitoring accuracy was low (.10 for students, −.02 for teachers) and did not differ between cue-prompt conditions. Regulation, indexed by the correlation between JOLs and restudy selections, was significant (−.38 for students, −.60 for teachers), but was also not affected by cue-prompt condition. In Experiment 2, teachers judged students’ comprehension of six texts without knowing the students’ names, so that only the keywords and summaries, not prior impressions, could inform judgments. Again, monitoring accuracy was generally low (.06), but higher for keywords (.23) than for summaries (−.10). These results suggest that monitoring intra-individual differences in students’ learning is challenging for teachers. Analyses of the diagnosticity and utilization of keywords suggest that these may contain insufficient cues for improving teacher judgments at this level of specificity.

## Introduction

Education increasingly relies on self-regulated study (Bjork et al. 2013). Nowadays, by the end of primary school, a substantial proportion of studying takes place outside the classroom. Within the classroom, too, children have a degree of autonomy in deciding what activities to perform and when to perform these activities. To become an effective self-regulated learner, a child needs to be able monitor his or her progress toward a given learning goal and set further study activities accordingly. However, even for seemingly simple tasks such as memorizing word pairs or pictures, 10-year-olds cannot reliably tell apart what they have and have not learned (e.g., Koriat and Shitzer-Reichert 2002; Schneider et al. 2000). Also, young children do not have the same tendency as adults to allocate study time to those items they have not yet learned (e.g., Dufresne and Kobasigawa 1989; Masur et al. 1973). Consequently, children’s decisions with regard to study activities are likely to lead to suboptimal outcomes. This is especially true for learning from text, where monitoring one’s progress has been argued to be inherently more challenging than for some other tasks (De Bruin et al. 2011; Redford et al. 2011). Thus, there is good reason to look for ways to help improve children’s regulation processes in this domain.

In this study we investigate a method for enabling primary school teachers to support children’s study regulation when learning from text, based on two so-called ‘cue-prompt’ strategies: keyword generation and summary writing. These strategies have been shown to improve both monitoring (i.e., discriminating what one has learned well enough from what one has not yet learned well enough) and control (i.e., making effective behavioral choices based on this information) for students who actively engage in them (De Bruin et al. 2011; Thiede and Anderson 2003). Our main research question was: Can keyword generation and summary writing improve the judgments of teachers who have access to the *products* (i.e., the keywords and summaries) of these strategies? We investigate these two strategies together, because their presumable costs and benefits differ in interesting ways: From the student’s perspective, generating keywords does not require much time and effort, as opposed to summarizing a text. From the teacher’s perspective, summaries potentiallyconstitute a richer set of cues about the student’s mastery of the material than keywords. In the following sections, we will discuss the foundations of comprehension judgments for teachers and students and then review the literature on keywords and summaries in more detail.

### Accuracy of teacher judgments

In general, teachers’ judgments in primary and secondary education are fairly accurate with regard to students’ performance (Südkamp et al. 2012). Judgment accuracy is usually operationalized as the correlation between the teacher’s judgment (whether this is a rating on a Likert-type scale, a grade equivalent, or the number of correct responses) and some type of objective measurement. One systematic review reports a median accuracy of .66 across studies in which a variety of judgments were compared against students’ achievement on a concurrently administered test (Hoge and Coladarci 1989). More recently, a meta-analysis of 75 studies found an overall correlation of .63 between teachers’ judgments and objective achievement measures (Südkamp et al. 2012).

In all of these studies, teachers provided a single judgment per student, or multiple judgments were aggregated to yield a single average judgment per student (but see Oudman et al. 2018, for a fine-grained analysis of item-specific judgments in mathematical understanding). As such, the literature is not informative with respect to teachers’ ability to distinguish, for a given student, between materials that the student has learned well and materials that the student has learned less well. Consider a child with average reading skills studying two texts to prepare for a test, one about Roman civilization and one about the climatic zones of the South-American continent. Depending on the difficulty of the texts and such factors as the student’s prior topic knowledge (Marr and Gormley 1982; McNamara and Kintsch 1996), mood (Bohn-Gettler and Rapp 2011; Bower et al. 1981), and motivational and attentional state at the time of reading (Unsworth and McMillan 2013), he or she will probably not learn equally much from each text. For teachers, being able to perceive such differences is necessary for providing adequate adaptive support and guiding students’ self-regulated learning (Van de Pol et al. 2011). Even though some authors have measured judgment accuracy by computing within-teacher correlations (e.g., Helmke and Schrader 1987; Thiede et al. 2015), we do not know of any published work that investigates whether teachers can accurately judge a given student’s understanding of one text relative to another. This intra-individual level of accuracy, however, is a common focus in research on students’ metacomprehension.

### Metacognitive accuracy in students’ self-regulated learning

In a typical metacomprehension experiment (e.g., Glenberg and Epstein 1985), participants read a number of texts to prepare for a test. Before taking the test, they are prompted to provide a judgment of learning (JOL) for each text, for example by rating their comprehension on a five-point scale. This allows the researcher to assess the agreement between the JOLs and actual test scores for each participant, usually by computing a Goodman-Kruskal gamma correlation (Goodman and Kruskal 1954). Two findings that have emerged from the literature on students’ metacomprehension are of importance here: first, monitoring accuracy (i.e., the extent to which one is able to discriminate between well-learned and less well-learned material) without a cue prompt is usually low. Gamma correlations of zero or lower have been reported for children (De Bruin et al. 2011, Redford et al. 2011) and of .27 for adults (Thiede et al. 2003). Second, monitoring accuracy in tasks that involve text comprehension can be improved by using delayed cue prompts, such as writing keywords or summaries. When students write a list of keywords that capture the essence of a text a sufficiently longtime after reading but prior to giving a JOL, their JOLs align with test performance markedly better. Gamma correlations increased to approximately .40 in sixth-graders (De Bruin et al. 2011) and .70 in adults (Thiede et al. 2003). Similar improvements in accuracy have been found when college students write summaries: Gamma correlations increased from .28 to .60 (Thiede and Anderson 2003) and from .21 to .64 (Anderson and Thiede 2008).

According to the cue-utilization framework (Koriat 1997), individuals base their judgment of how they will perform on a later memory test text on a variety of cues. These can be broadly classified as *intrinsic* cues (e.g., the a priori difficulty of the material), *extrinsic* cues (e.g., how long and deeply one has studied the material), and *mnemonic* cues (e.g., how easily the studied material comes to mind). For text comprehension, students have been shown to rely on their familiarity with the topic (Glenberg and Epstein 1985; Shanks and Serra 2014), how fluently they could process the text (Rawson and Dunlosky 2002; Thiede et al. 2010), and how quickly they access information in memory (Morris 1990). However, not all these cues are diagnostic of actual test performance, and may sometimes lead to over- or underestimation of test performance. An often-cited explanation for the effectiveness of delayed keyword and summary tasks is that both strategies make certain diagnostic mnemonic cues available to the learner. A likely diagnostic cue is the perceived ease with which the situation model of the text in long-term memory is accessed (Thiede et al. 2005).

### Keywords and summaries as predictors of test performance

Could delayed keywords and summaries similarly contribute to improving the accuracy of *teachers’* judgments? This seems reasonable, because both cue prompts result in a written product that provides the teacher with pointers toward students’ comprehension on a text-by-text level. For example, a keyword that matches one of the core concepts of a text might be an indicator of good comprehension, while a keyword that reflects a seductive yet unimportant detail may signal suboptimal comprehension. However, we know of no published attempt at uncovering the relation between keyword quality and text comprehension. Thus, it is as of yet unclear whether keywords are actually predictive of test performance. Research with college students suggests that it is the perceived fluency of the process of *generating* the keywords that underlies the improvement in monitoring accuracy (Thiede et al. 2005). Such differences in fluency may or may not be related to observable differences in keyword quality. One can imagine that an undergraduate student is able to come up with keywords that capture the most important concepts of a text, even if for some texts this takes more effort than others. For a sixth-grader, who generally has much less experience with reading and subsequently recalling texts, the quality of keywords might be more heterogeneous from one text to another. If not, then keywords are unlikely to lead to more accurate teacher judgments, because internal fluency information is available only to the student.

A different situation may apply for summaries. Research shows that summaries written by college students have diagnostic value when analyzed in a systematic way (Anderson and Thiede 2008; Thiede and Anderson 2003; Thiede et al. 2010). For instance, the number of ideas and semantic overlap with a gold standard summary both correlate significantly with test performance (Thiede and Anderson 2003). These cues might be relatively easy for teachers to pick up. Yet, while summary writing improves meta comprehension accuracy in college students, its effectiveness in primary education is less evident, for at least two reasons. First, primary school children often struggle to produce adequate summaries, recalling fewer idea units, showing less sensitivity to the importance of idea units, and using more words to express idea units than college students (Brown et al. 1983). If the summaries of a number of texts are equally difficult to write, regardless of how well the student has understood each text, then those summaries provide little basis to improve judgment accuracy on a text-by-text level. Second, even if some children have passed this bottleneck and produce summaries that are reflective of their level of understanding of each text, it is possible that this task is so cognitively demanding (cf. Roebers et al. 2007) that they are only marginally capable of utilizing these summaries in the service of comprehension monitoring. Teachers, on the other hand, only need to consult the summaries and should therefore have sufficient cognitive resources available to use the summaries to improve their judgments.

In sum, we propose that teachers can draw from different sets of cues when judging their students’ learning from text. First, teachers can use their knowledge of relatively stable student attributes and the difficulty of the materials, much like the intrinsic cues described by Koriat (1997). These are predictive of students’ overall performance relative to other students (Hoge and Coladarci 1989), but may have limited value for predicting intra-individual differences. Second, teachers can observe students’ behavior in the learning situation, such as whether a student spends enough time on a text to ensure deep encoding, similar to Koriat’s (1997) extrinsic cues. Third, within the context of this study, teachers can assess, in various ways, the quality of the keywords and summaries that students can produce from memory. These may reflect momentary fluctuations in students’ learning and therefore enhance the accuracy of teachers’ intra-individual judgments. Furthermore, it is likely that the more cues are available to the teacher, the more accurate teachers’ judgments should become, but not necessarily: Not all cues are equally predictive of test performance, and deciding which cues to use and how to weigh them is no trivial task (Kaiser et al. 2015). To better understand teachers’ monitoring, therefore, it is important to identify the cues that can help predict test performance and those that teachers use, and then determine to what extent these overlap.

### Regulation in students and teachers

While accurate monitoring is a necessary condition for successful self-regulated learning, it is by no means sufficient. We therefore also look at *control* (also referred to as *regulation*), the other component of metacognition (Nelson and Narens 1990). Importantly, more accurate regulation has been shown to result in better learning outcomes (e.g., Nelson et al. 1994; Rawson et al. 2011; Thiede et al. 2003). It is therefore interesting to investigate whether teachers can help students make more appropriate restudy selections. According to the discrepancy-reduction model (Dunlosky and Hertzog 1998), adults tend to restudy those materials they have learned least well after initial study (although different mechanisms may also guide study planning; see Metcalfe and Kornell 2005). This tendency seems to emerge around grade 5. In one study, 3rd-graders did not base their restudy choices on their JOLs at all when learning word pairs, but 5th-gradersdid, albeit inconsistently (Metcalfe and Finn 2013). Similarly, when learning from text, 4th-graders seem to make arbitrary restudy decisions. On the other hand, 6th-graders tend to select those texts for restudying for which they gave a lower JOL (De Bruin et al. 2011), but not to the same degree as adults in a similar paradigm (Thiede et al. 2003). It is not clear why children do not make optimal use of their monitoring. It has been suggested that accurately remembering the JOLs they gave earlier, especially when there are concurrent task demands, is beyond children’s cognitive capacity (Metcalfe and Finn 2013). That is, at the time children make their restudy selections, they do not always remember their JOLs or the metacognitive knowledge these JOLs were based on. Since such processing limitations apply less stringently to adults, we expect teachers to show better regulation than students.

Additionally, children’s metacognitive *experiences* (Efklides 2011) could complicate the relationship between JOLs and restudy selections. For example, children’s motivational and affective states (e.g., feeling of difficulty, appreciation of the text), which do not necessarily factor into JOLs, could favor or disfavor the selection of certain texts. These metacognitive experiences could thus prevent children from showing more rational regulation behavior. It is unlikely that teachers take such experiences into account when making restudy selections on behalf of their students. We therefore expect teachers to make a more straightforward coupling between JOLs and restudy selections.

To sum up, the aims of the present study are as follows. First, we investigate to what extent teachers can accurately judge intra-individual differences in their students’ learning from text and to what extent students’ keywords and summaries improve these judgments. Because summaries potentially contain the largest number of observable cues, we expect teachers’ judgment accuracy to be highest in the context of summaries, intermediate in the context of keywords, and lowest if there was no cue prompt. Second, we compare the effects of keyword generation and summary writing on monitoring accuracy in sixth-graders. Because writing summaries is highly cognitively demanding for children, we expect keywords to lead to better monitoring accuracy than summaries, which in turn should be more effective than no cue prompt. Third, we compare students and teachers with regard to regulation, expecting regulation to be better in teachers than in students. Finally, we explore to what extent the quality of keywords and summaries is predictive of test performance (*cue diagnosticity*) and to what extent teachers and students make use of this information (*cue utilization*).

To achieve these aims, we presented sixth-graders with six texts about various topics, asking them to provide delayed JOLs about each text before taking a test, either without a cue-prompt or after writing a list of keywords or a summary for each text. Subsequently, we also asked their teachers to provide a series of JOLs, using, if available, the keywords or summary that the student had written.

## Experiment 1

### Participants

Participants were 282sixth-graders (139 boys, 143 girls, ages 10.2–13.4 years, *M* = 11.84 years, *SD* = .54 years) and 14 teachers (3 males, 11 females) from 14 classes in 12 primary schools. All schools were located within the same urban area in The Netherlands. Students participated voluntarily and consent from parents was obtained prior to the study. Teachers had been teaching their current class for 9 months on average (range 1–34) and their average experience teaching in primary education was 16.8 years (range 6–31). The starting date for data collection differed for each class, running between November and March of the same school year.

The data of 27 students (10 in the no-cue condition, 7 in the keyword condition, and 10 in the summary condition) were incomplete, either because of computer failure or because the student was absent during the second testing day. Because this might lead to problems when computing and interpreting gamma correlations, these data were discarded. Unless otherwise specified, all analyses are based on the remaining 255 participants.

### Materials

#### Texts

The same six texts were used as in De Bruin et al. (2011). Texts were from the domain of biology (elephants, bears, and monkeys) and geography (Egypt, South-East Asia, and Mexico) and had an average length of 306 words (range 293–326 words). Previous testing verified that these texts had appropriate difficulty for use with sixth-graders (De Bruin et al. 2011). In addition, we included one shorter practice text (157 words) with questions to familiarize children with the procedure and the nature of the texts and questions.

#### Comprehension tests

The same comprehension tests were used as in De Bruin et al. (2011), consisting of five four-alternative multiple-choice questions for each text. The correct answers were mostly paraphrases of facts and opinions that were explicitly stated in the text (with the alternatives serving as plausible distracters) and in some cases required integration of information from different sentences. In general, the questions targeted ideas that were central to the text, rather than details. The answer options were displayed in randomized order.

Table 1 lists several characteristics for each comprehension test. Cronbach’s α for the individual comprehension tests ranged from .24 to .45 (*M* = .36, *SD* = .08), indicating low internal consistency for each comprehension test. This may indicate that separate test questions for each text did not measure a unidimensional construct. For example, the questions might measure memory for different facts stated in the text, which could be remembered independently from one another. The averaged item-rest correlations for the individual comprehension tests ranged from .11 to .24, (*M* = .18, *SD* = .05), suggesting that individual items, on average, discriminated fairly well. The mean performance per item across all comprehension tests was .60 (min = .33, max = .90, *SD* = .14). For two items, a distract or was selected more often than the correct answer. Because removing these items would invalidate the JOLs for the respective texts, these items were retained. The correlations between the total scores for each comprehension test and the summed totals for all other comprehension tests ranged from .36 to .55, (M = .46, *SD* = .07), suggesting that for each text, students who did well on that comprehension test also tended to do well on the other tests.

**Table 1:** Internal consistency, item-rest correlations, item difficulty, and correlation with other tests for each comprehension test

		Item-rest correlation			Item difficulty			
Text	Cronbach’s α	*M*	min	max	*M*	min	max	Correlation with other texts
Elephants	.24	.11	−.01	.18	.52	.33	.69	.52
Bears	.32	.15	.12	.21	.54	.34	.76	.36
Monkeys	.36	.19	.11	.27	.65	.41	.90	.49
Egypt	.37	.18	.14	.29	.64	.57	.72	.47
South-East Asia	.44	.23	.11	.34	.57	.45	.73	.38
Mexico	.45	.24	.11	.30	.63	.46	.76	.55

### Design and procedure

The teachers received a copy of the texts and test questions, which they were asked to read prior to the start of the experiment. They were told that they could consult their copy at any time during the experiment. Children performed the experiment individually on a personal computer in or just outside the classroom; the exact placement differed per school. Some teachers chose to let more than one child work on the task at the same time, if more computers were available.

Because reading all six texts in one sitting would stretch beyond children’s attentional capacities, the experiment was divided into two sessions. In the first session, children were randomly assigned to a condition (no-cue, keyword, or summary) and a text set (animals or geographical regions). The order in which the texts were presented was varied according to a Latin-square design, resulting in three different lists per session. Sessions were approximately 1 week apart.

The experiment was presented via Qualtrics (www.qualtrics.com) in a full-screen browser window. All texts, cue prompts and JOL prompts were presented on separate pages. It was not possible to browse back through the experiment at any time. In the first session, children received written instructions and exercised the procedure with the practice text. In the second session, children started directly with studying the experimental texts. The anticipated duration was 20–25 min for the student part of session 1, 15–20 min for the student part of session 2, and 2 min per student for the teacher part of both sessions. Table 2 provides a schematic overview of the experimental procedure.

**Table 2:** Overview of the study procedure for a given session*.* ‘S-JOL’ refers to a judgment of learning provided by the student. ‘T-JOL’ refers to a judgment of learning provided by the teacher

	No cue	Keyword	Summary
Student part	Read text 1 Read text 2 Read text 3	Read text 1 Read text 2 Read text 3	Read text 1 Read text 2 Read text 3
	S-JOL text 1 S-JOL text 2 S-JOL text 3	Write keywords text 1 S-JOL text 1 Write keywords text 2 S-JOL text 2 Write keywords text 3 S-JOL text 3	Write summary text 1 S-JOL text 1 Write summary text 2 S-JOL text 2 Write summary text 3 S-JOL text 3
	Restudy choices	Restudy choices	Restudy choices
	Test	Test	Test
Teacher part	T-JOL text 1 T-JOL text2 T-JOL text 3	Read keywords text 1 T-JOL text 1 Read keywords text 1 T-JOL text 2 Read keywords text 3 T-JOL text 3	Read summary text 1 T-JOL text 1 Read summary text 2 T-JOL text 2 Read summary text 3 T-JOL text 3
	Restudy choices	Restudy choices	Restudy choices

#### Text study

Students read the three experimental texts at their own pace. All texts were displayed in black 14-point Arial font against a white background.

#### Cue prompt

In the keyword condition, students saw the title of the text and were prompted to provide a list of up to five keywords that they thought were the most important words from that text. In the summary condition, students were prompted to write a summary that captured the most important information in the text. Titles of the texts were shown in the order in which they had been read.

#### Student JOLs

Student JOLs (S-JOLs) per text were prompted on a 6-point scale (range 0–5) with the question “How many questions about the text ‘[text title]’ do you think you will answer correctly?” displayed above.

#### Student restudy selections

Students were shown the titles of the three texts they had just read and asked to indicate which text(s) they wanted to restudy by checking the boxes below the titles. Prior to making their choice, students were informed that there would be no opportunity for actual restudying. We chose this approach because actually restudying texts would distort the relation between JOLs and test performance (Kimball and Metcalfe 2003).

#### Test taking

Students answered five four-alternative multiple choice questions per text. Students could only proceed to the next page after answering all questions. When students were done, they were shown a message to thank them for their participation and to instruct them to leave the browser window open, so that their teacher could answer some questions.

#### Teacher JOLs

Teacher JOLs (T-JOLs) were prompted using the same 6-point scale as S-JOLs, with the question “How many questions about the text ‘[text title]’ do you think [name of student] has answered correctly?”. If the student had written keywords or a summary, these were displayed above the JOL prompt in italics.

#### Teacher restudy selections

As in the student restudy selection phase, the teacher saw the titles of the texts that the student had read and was asked to select 0–3 texts that the student should restudy by checking the respective boxes.

### Data analysis

As in previous research, we defined monitoring accuracy as the intra-individual gamma correlation between text-by-text JOLs (0–5) and text-by-text test scores (0–5). Regulation was defined as the intra-individual gamma correlation between text-by-text JOLs (0–5) and text-by-text restudy selections (0 or 1).^1^ Thus, a stronger negative correlation indicates more accurate regulation.

We defined cue diagnosticity as the intra-individual gamma correlation between the frequency of a particular type of idea unit in the keywords and summaries (e.g., main idea, detail; see ‘Coding of keywords and summaries’) and test performance. Cue utilization was defined as the intra-individual gamma correlation between the frequency of a particular type of idea unit and JOL magnitude.

#### Statistical models

Even though the data have a hierarchical structure (i.e., gamma correlations at the student level are clustered under teachers), we did not use hierarchical linear modeling because of the relatively small number of clusters at level-2 (the teacher or classroom level), which might cause the standard errors of the level-2 variance components to be biased (McNeish and Stapleton 2016). Instead, for the analyses of the effect of cue-prompt on students’ monitoring and regulation (and other student-level variables in control analyses), we used ANCOVA, removing the intercept from the model and including classrooms as dummy-coded covariates to model cluster affiliation (following the recommendations in McNeish and Kelley 2018). For the analyses of teachers’ monitoring and regulation, we computed, for each teacher, the means over the gamma correlations for all students in a given cue-prompt condition. The condition means were then compared using repeated-measures ANOVA.

#### Coding of keywords and summaries

An independent rater scored all keywords in the dataset according to a four-category rubric, similar to the coding scheme used by Van Loon et al. (2014) (examples pertaining to the text about monkeys): main idea (e.g. ‘experiments’), detail (e.g., ‘smoking’), incorrect (e.g., ‘extinct’), or commission (e.g., ‘Africa’). The rater also parsed all summaries into idea units and scored these using the same rubric. A second independent rater then scored the keywords for all texts from 15 randomly selected students and the summaries from another 15 students. Raters agreed on the coding of 86% of the keywords; Cohen’s κ was .77, *p* < .001. Raters agreed on the coding of 68% of the idea units in summaries; Cohen’s κ was .57, *p* < .001. Because inter-rater agreement for summaries was low, the coding instructions were revised and the raters (of which one was new) scored all summaries again. Raters now agreed on the coding 66% of the units; Cohen’s κ was .52, *p* < .001. We conclude that the coding strategy for summaries was problematic and therefore drop the content analysis of the summaries. At the same time, the low reliability may be a meaningful statistic, because it suggests that even for instructed raters without time pressure, the summaries in this sample were difficult to judge unambiguously against a given standard.

## Results

Table 3 provides the descriptive statistics of test scores, JOLs, restudy selection probabilities, and the gamma correlations that were derived from these measures. To make sure that any effects of cue-prompt condition were located at the meta comprehension level, rather than the comprehension level, we evaluated the effect of cue-prompt on test performance. This effect was significant, *F*(2, 239) = 4.741, *p* = .010. Post-hoc comparisons with Bonferroni correction indicated that test scores in the no-cue condition were higher than in the summary condition, *Mdiff =* .364, *SE* = .119, *p =* .007. However, while this effect was significant, it was small on a 0–5 scale, and therefore does not compromise conclusions about monitoring.

**Table 3:** Means and standard deviations of test scores, judgments of learning, restudy selection probabilities, monitoring accuracy, and regulation by cue prompt in experiment 1

	No cue	Keyword	Summary
Test score	3.16 (.78)	3.02 (.83)	2.82 (.79)
Judgment of learning			
Teachers	2.99 (.98)	2.92 (.98)	3.03 (.97)
Students	3.17 (.82)	2.76 (.91)	3.02 (.81)
Restudyselectionprobability			
Teachers	.41 (.28)	.43 (.27)	.42 (.23)
Students	.48 (.24)	.50 (.24)	.45 (.24)
Monitoring accuracy			
Teachers	−.09 (.60)	.00 (.66)	.04 (.63)
Students	.17 (.65)	.09 (.62)	.05 (.67)
Regulation			
Teachers	−.70 (.52)	−.60 (.59)	−.74 (.55)
Students	−.41 (.68)	−.34 (.74)	−.39 (.73)

Test scores and judgments of learning were measured on a scale of 0–5. Restudy selection probability was measured categorically as 0 (not selected) or 1 (selected)

Monitoring accuracy and regulation are intra-individual gamma correlations that range from −1 to 1. For monitoring, 1 indicates perfect accuracy; for regulation, −1 indicates perfect accuracy.

### Monitoring accuracy in teachers and students

#### Teacher monitoring accuracy

For 23 students (11 in the no-cue condition, 5 in the keyword condition, and 7 in the summary condition), we could not compute a gamma correlation because of invariance in T-JOLs. The grand mean of the model was −.016, 95% CI [−.113 .082], suggesting that teachers’ monitoring accuracy was not better than chance. The effect of cue prompt was not significant, *F*(2, 26) = 2.586*, p* = .095, suggesting that teachers’ monitoring accuracy did not differ between cue-prompt conditions.^2^

#### Student monitoring accuracy

For 26 students (9 in the no-cue condition, 4 in the keyword condition, and 13 in the summary condition) we could not compute a gamma correlation because of invariance in S-JOLs. The grand mean of the model was .103, 95% CI [.020 .186], suggesting that students’ monitoring accuracy was better than chance. The effect of cue prompt was not significant, *F*(2, 213) = .685, *p* = .505, suggesting that monitoring accuracy did not differ across conditions.

### Regulation in teachers and students

#### Teacher regulation

For 58 students (27 in the no-cue condition, 21 in the keyword condition, and 10 in the summary condition) we could not compute gamma correlations because of invariance in T-JOLs or restudy selections. As a result, data from one teacher could not be included in the analyses. The grand mean of the model was −.635, 95% CI [−.776 –.495], suggesting that overall, teachers were more likely to select texts for restudying which they had given lower T-JOLs. The effect of cue-prompt was not significant, *F*(2, 24) = 1.617, *p* = .219, suggesting that teachers’ regulation did not differ between cue-prompt conditions.

#### Student regulation

For 53 students (18 in the no-cue condition, 15 in the keyword condition, and 21 in the summary condition) we could not compute gamma correlations because of invariance in S-JOLs or restudy selections. The grand mean of the model was −.379, 95% CI [−.480 –.277], suggesting that students were more likely to select texts for restudying which they had given lower S-JOLs. The effect of cue prompt was not significant, *F*(2, 186) = .104, *p* = .901, suggesting that students’ regulation did not differ between cue-prompt conditions.

To compare teachers’ and students’ regulation we ran an independent samples *t*-tests (equal variances not assumed) on the gamma correlations for each classroom (i.e., students’ gamma correlations were aggregated at the classroom level to account for dependencies). Regulation was significantly higher for teachers than for students, *Mdiff* = .253, *t*(26) = 3.227, *p* = .004.

### Cue diagnosticity andcue utilization

On average, students wrote 4.37 keywords per text (*SD* = 1.12). Table 4 lists the average number of each type of keyword, their diagnosticity, and their utilization by students and teachers. None of the cues were diagnostic, including the number of main ideas. Students and teachers utilized this cue, however, as indicated by correlations of .24 and .18, respectively. Furthermore, textual details were significantly correlated with S-JOLs (.23) and incorrect idea units were significantly correlated with T-JOLs (.27).

**Table 4:** Means and standard deviations of the number, diagnosticity, teacher utilization, and student utilization of keywords by type

	Main idea	Detail	Incorrect	Commission
*N*	2.17 (1.36)	1.31 (1.11)	.30 (.59)	.59 (.97)
Diagnosticity	.12 (.60)	.00 (.61)	−.07 (.74)	−.14 (.76)
Student utilization	.24 (.56)***	.23 (.63)**	.19 (.78)	−.16 (.78)
Teacher utilization	.18 (.71)*	.07 (.66)	.27 (.81)*	−.12 (.76)

* denotes statistical significance at the .05 level. ** denotes statistical significance at the .01 level. *** denotes statistical significance at the .001 level

Possibly, students who accurately monitored their learning were the ones that generated the most diagnostic keywords. To further explore this relationship, we computed cue diagnosticity for the keywords written by the subset of students whose monitoring accuracy was .5 or higher (*n* = 24). The pattern of results was nearly identical to the overall sample. Main ideas and errors of commission were nominally more diagnostic (.25 and − .24, respectively) than in the overall sample, but these correlations were not statistically significant.

## Discussion

Based on the literature on teacher judgments and metacognitive monitoring, we expected teachers’ judgments of students’ comprehension to improve when these judgments could be informed by keywords or summaries. However, teachers’ monitoring accuracy was generally low and did not improve with either cue-prompt. Closer analyses of the students’ keywords revealed that their quality did not predict intra-individual variation in test performance. Thus, it is not surprising that these did not improve teachers’ monitoring accuracy.

The second goal was to compare the effect of keyword generation and summary writing on sixth-graders’ comprehension monitoring. Given that the materials and procedures for the keyword condition were adopted from de Bruin et al. (2011, Experiment 2), it is surprising that students’ own monitoring accuracy was not closer to the level observed in that study(.42 in 6th-graders). It should be noted that other outcomes were similar in both studies: Test performance in the keyword condition was slightly higher and less variable in our study (*M* = 3.02, *SD* = .83vs. *M* = 2.65, *SD* = .98), while S-JOLs were somewhat lower and more variable (*M* = 2.76, *SD* = .91 vs. *M* = 3.21, *SD* = .80). Thus, it is the relative monitoring accuracy, independently of other aspects, that differed between the studies. One reason might be that sixth-graders utilized two types of non-diagnostic cues, namely the number of main ideas and details. In the General Discussion we review some further explanations for this discrepancy.

With respect to the third goal, comparing students’ and teachers’ regulation, the results supported our expectations. Both teachers and students were more likely than chance to select those texts for restudy for which they had given lower JOLs, with teachers showing a stronger correlation (−.64 vs. −.38). Without accurate monitoring preceding these restudy selections, however, it is unlikely that learning outcomes will improve. In line with this, the correlation between restudy selections (both by teachers and students) and test performance was not better than chance.

The fourth goal was to systematically explore the diagnosticity and utilization of keywords and summaries. It was not possible to determine the diagnosticity of summaries, while for keywords we found that their quality did not predict intra-individual variation in test performance. Arguably, our analyses might have underestimated the diagnosticity of the keywords. Even though the categories in our coding scheme show considerable overlap with those used by Anderson and Thiede (2008; gist and details) and Van Loon et al. (2014; correct relations, factual information, omissions, and commissions), our coding scheme might not have succeeded in making certain latent cues (e.g., completeness of the summary, combinations of keywords) manifest. Teachers’ self-reported interpretation and utilization of keywords and summaries may provide some insights in this matter and suggest new ways of looking at these student products.

The cue utilization analyses suggest a number of reasons for the low monitoring accuracy in teachers. First, incorrect keywords were associated with higher T-JOLs. We would have expected teachers to identify these keywords as misrepresenting ideas expressed in the text and therefore give lower T-JOLs. Possibly, then, teachers did not always carefully assessthe keywords or did not have an optimal mental representation of each text themselves. Second, teachers’ knowledge of the students’ general aptitude and past performance on related tasks may have influenced their judgments (cf. Kaiser et al. 2015; Oudman et al. 2018). It is conceivable that teachers relied more on this knowledge than on the summaries or keywords at hand when providing a T-JOL.

In Experiment 2, we attempt to enhance our understanding of teachers’ intra-individual judgments in two ways. First, we explore the effect of keywords and summaries on monitoring accuracy when students’ names are blinded from the T-JOL prompts. Second, we explore teachers’ conscious utilization of the cues in keywords and summaries and its relation with monitoring accuracy by asking them to retrospectively report on what aspects of the keywords and summaries they paid attention to when judging students’ text comprehension.

## Experiment 2

### Participants

Participants were 15 teachers (10 females), whose average primary school teaching experience ranged from 5 to 32 years (*M* = 14.0) and their average teaching experience with sixth-graders ranged from 0 to 12 years (*M* = 4.6 years). Data collection ran in May and June of one school year and August and September of the next.

### Materials

We randomly sampled 10 students from the keyword condition and 10 students from the summary condition in Experiment 1, with the only constraint that these students showed variance in their test scores. The keywords or summaries that the student had written for each text in Experiment 1 were displayed to the participating teachers without revealing the name of the student. On average, keyword lists contained 4.13 keywords (*SD* = 1.20) and summaries contained 30.32 words (*SD* = 14.92). For this sample, average comprehension test performance was 3.18 (*SD* = .65) in the keyword condition and 2.93 (*SD* = .52) in the summary condition.

### Design and procedure

Teachers were informed about the goal of the study (to investigate how well they could judge students’ text comprehension based on keywords and summaries) and received a general overview of the procedure. After that, they reviewed the six texts and the comprehension questions. To replicate the procedure of Experiment 1 as much as possible, they then provided JOLs for keywords and summaries in an intermixed fashion. The order of students was randomized across teachers, but the order of the texts was the same for each student. JOLs were provided on a scale of 0–5, with the prompt “How many questions about the text [text title] do you think student [*n*] has answered correctly?”. The keywords or the summary was displayed above the JOL prompt in italics. Each JOL was provided on a separate page. After each student, a screen flashed for 2 s to indicate that participants were about to give JOLs for a new student. After they had completed all JOLs, teachers were prompted to describe in two text boxes, retrospectively for the entire experiment, what aspects of the keywords and summaries they had taken into account when giving their judgments.

### Results

Test scores, T-JOLs, and the intra-individual gamma correlations betweenthese variables are listed in Table 5. Because T-JOLs were strikingly lower than in Experiment 1, we ran two independent samples *t*-tests (equal variances not assumed) to compare the means for both conditions across experiments. The difference was statistically significant, both for keywords, *Mdiff* = −.78, *t*(27) = −3.294, *p* = .001, and summaries, *Mdiff* = −1.25, *t*(27) = −6.069, *p* < .001.

**Table 5:** Means and standard deviations of test scores, teachers’ judgment of learning, and monitoring accuracy by cue prompt in experiment 2

	Keyword	Summary
Test score	3.18 (.65)	2.93 (.52)
Judgment of learning	2.24 (.34)	1.78 (.46)
Monitoring accuracy	.23 (.11)	−.10 (.10)

Test scores were derived from Experiment 1

#### Teachers’ monitoring accuracy

We performed a repeated measures ANOVA with cue prompt (keywords vs. summaries) as independent variable and monitoring accuracy as dependent variable. The grand mean of the model was .063, 95% CI [.027 .098], indicating that overall, teachers’ monitoring accuracy was better than chance. The effect of cue-prompt condition was significant, *F*(1, 14) = 58.524, *p* < .001, suggesting that teachers’ monitoring accuracy was greater in the keyword condition than in the summary condition.

##### Self-reported cue utilization

Teachers’ self-reports regarding keyword and summary utilization were coded inductively. All teachers mentioned between two and five attributes they paid attention to. For keywords, seven attributes were mentioned by at least two teachers; for summaries, five attributes were mentioned by at least two teachers. These are displayed in Table 6. For keywords, the most frequently mentioned attributes were main ideas (*n* = 9), number of keywords (*n* = 5), and details (*n* = 4). For summaries, the most frequently mentioned attributes were main ideas (*n* = 10), details (*n* = 6), length (*n* = 4), and whether the information occurred in the text (*n* = 5). All teachers who mentioned details referred to these in contrast to main ideas (but not all teachers who mentioned at main ideas also referred to details) so it can be assumed that they interpreted the occurrence of details as a sign of poor comprehension. Only one teacher mentioned looking at whether the keywords and summaries would be helpful for answering the test questions. Also, three teachers paid attention to spelling when looking at keywords, but did not mention this for summaries.

**Table 6:** Attributes of keywords and summaries that teachers reported taking into account when judging text comprehension

	Keywords		Summaries	
Attribute	*n*	Correlation with monitoring	*n*	Correlation with monitoring
Main ideas	9	−.39, n.s.	10	.25, n.s.
Details	4	−.33, n.s.	6	.28, n.s.
Pertains to text	2	.22, n.s.	5	−.48, n.s.
Number	5	−.26, n.s.	–	–
Length	–	–	4	−.41, n.s.
Spelling	3	.06, n.s.	0	–
Sentence structure	–	–	3	−.08, n.s.
Each paragraph covered	2	−.14, n.s.	0	–
Other	3	–	3	–

‘n.s’ means not significant at α = .05.‘–’ signifies that the attribute did not apply to either summaries or keywords. ‘0’ signifies that no mentions of that attribute were observed

To explore whether any of the attributes that teachers reported paying attention to were systematically associated with monitoring accuracy, we computed point-biserial correlations for each of the attributes (0 = not used, 1 = used) with the teachers’ monitoring accuracy. The correlations are reported in Table 6. None of the attributes were significantly correlated with monitoring accuracy, all *p*s > .081.

## Discussion

Teachers judged intra-individual differences in learning from text in anonymous students with above-chance accuracy. This was mainly driven by the accuracy in the keyword condition, which was significantly higher than in the summary condition. Teachers’ self-reported cue utilization did not reveal any striking differences between keywords and summaries: In both cases, the most frequently mentioned attribute was the presence of main ideas from the text, followed by the presence of details from the text and the number of keywords or length of the summary. Thus, it seems that teachers’ conscious utilization of keywords and summaries is highly similar. Given that we could not reliably assess whether keywords and summaries differ in the extent to which they contain cues that are diagnostic of test performance, we can only speculate about the origin of this difference in monitoring accuracy. While both the keyword and the summary group were representative of the larger sample in Experiment 1 in terms of test performance, there might have been a larger contrast in the quality of their keywords and summaries. A design in which each teacher sees the products of a different subset of students could enhance the comparability with Experiment 1.

## General discussion

The main research question of this study was whether two cue-prompt strategies, keyword generation and summary writing, would improve the accuracy of teachers’ intra-individual judgments of students’ learning from text. The focus was on relative accuracy, operationalized as the gamma correlation between text-by-text judgments and text-by-text test scores. To our knowledge, no study has looked at this type of teacher judgments, even though discrimination of what a given student has learned well from what he or she has learned less well is an important basis for adaptive individual support (Van de Pol et al. 2011).

In the no-cue condition of Experiment 1, teachers did not accurately predict the differences in their students’ text-by-text test performance. This seems to contrast with findings from earlier studies, as summarized in Südkamp et al. (2012). In these studies, however, judgment accuracy was operationalized as teachers’ ability to predict differences in test performance between students, rather than within students. Making intra-individual judgments is a process that differs qualitatively from making inter-individual judgments. The teacher does not only need to know the relative difficulty of each text, but also their relative difficulty *for each student*. For instance, while a given student might struggle with reading comprehension in general, he or she might also have a keen interest in geography, making it likely that he or she learns relatively much from texts about this topic. In addition to relatively stable student characteristics, such as general aptitude and topic interest, there can be fluctuations on smaller time scales that influence students’ learning outcomes, such as mood, time on task, and various sorts of distractions. Providing accurate intra-individual judgments might require the teacher to attend to all these factors, but it is questionable if all of these factors are directly observable. As such, it is not surprising that accuracy for this type of judgment is lower than that of inter-individual judgments.

All the same, if the factors outlined above determine students’ performance on a comprehension test, they can also be expected to influence the quality of the keywords and summaries that students write at a delay after reading. We reasoned that the keywords and summaries should serve as externalizations of students’ long-term memory representations of the texts, thus making potentially diagnostic cues available (Koriat 1997). Contrary to our expectations, when teachers consulted these products prior to providing judgments, the accuracy of their judgments did not improve. Further analyses revealed that keyword quality was not predictive of test performance and that children’s summaries were difficult to interpret in the first place. This is consistent with the idea that delayed cue-prompts are effective because not what students write, but the internal feedback they receive in the process provides them with diagnostic cues (Thiede et al. 2005). By definition, these cues are only available to the student, not to the teacher.

There could also be other, task-related explanations for not replicating the keyword effect. In Experiment 1, some experimental control was deliberately waived to make the task as reflective of daily classroom practice as possible. Teachers gave their JOLs while having observed some students at work but having been more distracted at other moments. This ‘noise’ is a hallmark of everyday classroom situations and may cause even very robust learning strategies, such as retrieval practice, to not always yield practical advantages in natural settings (Goossens et al. 2014). In Experiment 2, some external variables were more closely controlled: Teachers did not have any information about the students except for the keywords and summaries they had written, and performed the task in a more quiet setting. In this situation, teachers’ monitoring accuracy was better than chance when students had written keywords (.23), but still not at a level which sixth-graders have been able to achieve by themselves (.42; De Bruin et al. 2011). Given that our materials and procedures were adapted from that study, it is important to consider what might have caused this difference.

First, in our experiment students performed the experimental tasks on a computer rather than with pencil and paper. Metacognitive monitoring during on-screen learning has been found to be worse than during on-paper learning (Ackerman and Goldsmith 2011). Second, students did not perform filler tasks between the reading of each successive text, whereas De Bruin et al. (2011) used a short drawing task for students who finished reading early. Given that the delay between learning and judgment is a crucial determinant of metacomprehension accuracy (e.g., Dunlosky and Nelson 1992; Nelson and Dunlosky 1991), such timing differences may have affected metacomprehension accuracy. Third, there was no lag between keyword generation and JOL (K1-JOL1, K2-JOL2, K3-JOL3), whereas students in De Bruin et al. (2011) first generated keywords for all texts and then provided JOLs (K1-K2-K3, JOL1-JOL2-JOL3). Thus, the design in De Bruin et al. (2011) may have facilitated relative comparisons between texts. Research with college students, however, has contrasted these exact conditions and found no difference in metacomprehension accuracy (Thiede et al. 2005). Still, it is possible that children profit from a procedure that facilitates relative comparison more than college students. In general, the metacognitive benefits for children of generating keywords may be moderated by several variables, warranting further research on this topic.

The questions we used to measure text comprehension also prompt discussion. Most of these questions targeted the text-base level of representation, rather than the situation model (Graesser et al. 1997). This matched the nature of the texts, which focused on thematically related facts and included few causal relations. Previous research has shown that generating keywords improves monitoring accuracy for detail tests, albeit to a lesser extent than inference tests (Thiede et al. 2012). A crucial factor here is whether the type of test students are expecting matches the actual test (Thiede et al. 2011; Thomas and McDaniel 2007). In our study children practiced the reading-judgment-test taking routine prior to reading the critical texts. It is therefore unlikely that their low monitoring accuracy is caused by comprehension test expectations. Nevertheless, it would be worthwhile to test if students’ and teachers’ monitoring accuracy improves when the test consists of inference questions, which is what one would expect if the cues generated during a delayed keyword or summary task are based on the situation model of the text, rather than a more superficial level of representation (Thiede et al. 2005).

Furthermore, the multiple choice format may have worked against the keyword and summary tasks showing their full potential for improving monitoring accuracy. While the comprehension tests had some desirable properties (e.g., most questions had adequate difficulty, and no floor or ceiling effects were observed), their internal consistency was low, with Cronbach’s α ranging from .24 to .45. Obviously, the more measurement error a test contains, the more difficult it becomes to predict one’s test performance, regardless of one’s metacognitive ability. This may have limited the degree to which not only JOLs, but also keyword characteristics correlated with test performance. Furthermore, writing keywords and summaries requires a retrieval process, while multiple choice questions typically prompt a recognition response. Consequently, the cues that are generated during the keyword and summary tasks may not be diagnostic ofstudents’ ability to respond correctly to the questions during test-taking. However, it is not clear if this match between response types in the JOL phase and during test-taking is critical for the keyword effect to occur, as previous demonstrations of the keyword effect also used multiple-choice questions (e.g., De Bruin et al. 2011; Thiede et al. 2003, 2005). Besides, the particular combination of content (factual information) and format (multiple choice) that was used in the present studyis common in primary school curricula. Even if our finding that keyword generation and summary writing have no effect on teachers’ monitoring only generalizes to factual questions in a multiple-choice format, this still has a clear practical counterpart.

The sample of teachers in this study was rather heterogeneous, with years of service in primary education ranging from 6 to 31 years. Even though this range does not include absolute novices, there might be considerable differences in experience. Previous research, however, did not find an association between teacher experience and item-specific judgment accuracy (Impara and Plake 1998). Also, some teachers had known their class for more than a year, whereas others had been teaching their class for no more than a month. This state of affairs may have contributed to variation in the accuracy of teacher judgments in Experiment 1, but was irrelevant to Experiment 2, where student products were anonymized. To investigate the possible role of teaching experience and familiarity with the students on intra-individual judgment accuracy, studies with a higher degree of experimental control and more statistical power at the teacher level are needed.

The verdict on the feasibility of summary writing as a metacognitive strategy for sixth-graders is less equivocal. Summaries did not improve monitoring accuracy compared tothe other conditions in Experiment 1 and led to lower monitoring accuracy than keywords in Experiment 2. Also, writing summaries was associated with lower test scores in Experiment 1, possibly because there was a longer delay between reading and test-taking compared to the other conditions, or because the task imposed a higher cognitive load. This pattern contrasts with the metacognitive benefits of summary writing that have been found for adults (Anderson and Thiede 2008; Thiede and Anderson 2003), but can be explained in the light of earlier research which shows that fifth- and seventh-graders write summaries less efficiently and with less sensitivity to the importance of ideas than college students (Brown et al. 1983).

To conclude, in this study we explored a new approach to facilitating metacognitive monitoring by focusing on teachers’ text-by-text judgments of students’ comprehension. This approach was tested in the classroom, allowing us to directly evaluate its effects in a natural setting. As previous studies have shown that monitoring comprehension of multiple texts is difficult for children and adolescents, the present study suggests that it is no less difficult for teachers to do it for them. The fact that the same teachers’ inter-individual judgments were nearly as accurate those reported in earlier studies (Südkamp et al. 2012) suggests that it is the task of providing intra-individual judgments itself that is challenging. While the present results surprisingly do not show the expected benefits of the cue prompt strategies, teachers were reliable when it came to making restudy decisions based on their judgments. If future research can separate some diagnostic signal from the noise of student-generated keywords and summaries, these could still provide the key to a low-effort method that enables teachers to help students better regulate their learning.

## Footnotes

1 Alternatively, one could define regulation as the correlation between test performance and restudy selections. This gives insight into whether teachers and students actually selected those texts for restudy for which performance was lower. For teachers, the intra-individual gamma correlations between test performance and restudy selections were .05 (*SD* = .31), .04 (*SD* = .46), and − .04 (*SD* = .29) for the no-cue, keyword, and summary conditions, respectively; for students, the correlations were − .06 (*SD* = .62), −.03 (*SD* = .69), and − .10 (*SD* = .71). Neither for teachers nor students did these correlations differ significantly from each other, *F*s < 1.

2 To check whether teachers’ inter-individual judgment accuracy was comparable to earlier studies, we computed Pearson correlations between each teacher’s average T-JOL for a given student and that student’s average test performance. The coefficients of these inter-individual correlations were .52 (*SD* = .31) for the no-cue condition, .64 (*SD* = .24), for the keyword condition, and .43 (*SD* = .51) for the summary condition. We further analyzed these correlations in the same way as teachers’ monitoring and regulation. The grand mean of the model was .528, 95% CI [.377 .679], suggesting that overall, teachers’ inter-individual judgments were more accurate than chance. The effect of cue-prompt was not significant, *F*(2, 24) = 1.288, *p* = .294, suggesting that inter-individual judgment accuracy did not differ between cue-prompt conditions.
